# Endothelial NOX5 Expression Modulates Thermogenesis and Lipolysis in Mice Fed with a High-Fat Diet and 3T3-L1 Adipocytes through an Interleukin-6 Dependent Mechanism

**DOI:** 10.3390/antiox11010030

**Published:** 2021-12-24

**Authors:** Jorge G. García, Carlos de Miguel, Fermín I. Milagro, Guillermo Zalba, Eduardo Ansorena

**Affiliations:** 1 Department of Biochemistry and Genetics, University of Navarra, 31008 Pamplona, Spain; jgarcia.51@alumni.unav.es (J.G.G.); cdmiguel@unav.es (C.d.M.); gzalba@unav.es (G.Z.); 2 Navarra Institute for Health Research (IdiSNA), 31008 Pamplona, Spain; fmilagro@unav.es; 3 Center for Nutrition Research, Department of Nutrition, Food Science and Physiology, University of Navarra, 31008 Pamplona, Spain; 4 Centro de Investigación Biomédica en Red Fisiopatología de la Obesidad y Nutrición (CIBERobn), Instituto de Salud Carlos III, 28029 Madrid, Spain

**Keywords:** obesity, NADPH oxidase 5, thermogenesis, lipolysis, IL-6

## Abstract

Obesity is a global health issue associated with the development of metabolic syndrome, which correlates with insulin resistance, altered lipid homeostasis, and other pathologies. One of the mechanisms involved in the development of these pathologies is the increased production of reactive oxygen species (ROS). One of the main producers of ROS is the family of nicotinamide adenine dinucleotide phosphate (NADPH) oxidases, among which NOX5 is the most recently discovered member. The aim of the present work is to describe the effect of endothelial NOX5 expression on neighboring adipose tissue in obesity conditions by using two systems. An in vivo model based on NOX5 conditional knock-in mice fed with a high-fat diet and an in vitro model developed with 3T3-L1 adipocytes cultured with conditioned media of endothelial NOX5-expressing bEnd.3 cells, previously treated with glucose and palmitic acid. Endothelial NOX5 expression promoted the expression and activation of specific markers of thermogenesis and lipolysis in the mesenteric and epididymal fat of those mice fed with a high-fat diet. Additionally, the activation of these processes was derived from an increase in IL-6 production as a result of NOX5 activity. Accordingly, 3T3-L1 adipocytes treated with conditioned media of endothelial NOX5-expressing cells, presented higher expression of thermogenic and lipolytic genes. Moreover, endothelial NOX5-expressing bEnd.3 cells previously treated with glucose and palmitic acid also showed interleukin (IL-6) production. Finally, it seems that the increase in IL-6 stimulated the activation of markers of thermogenesis and lipolysis through phosphorylation of STAT3 and AMPK, respectively. In conclusion, in response to obesogenic conditions, endothelial NOX5 activity could promote thermogenesis and lipolysis in the adipose tissue by regulating IL-6 production.

## 1. Introduction

Obesity is characterized by an increase in body weight gain derived from an imbalance between energy intake and energy expenditure. Normally, a disruption in energy balance evolves in fat accumulation and alteration of adipose tissue homeostasis [1]. As a result, obesity frequently leads to the appearance of other pathologies and metabolic alterations such as insulin resistance, type 2 diabetes, hypertension, and cardiovascular disorders [2].

Humans possess two main types of adipose tissue. White adipose tissue (WAT) is the most abundant, and its main function is to store the excess of energy from food as triglycerides [3]. Brown adipose tissue (BAT) is less abundant and is characterized by transforming energy into heat [4], presenting high mitochondrial density and increased energy expenditure by promoting heat production inside these organelles. This process is known as thermogenesis and is regulated by proliferator-activated receptor γ coactivator 1α (*Pgc1α*), which is considered the master regulator of mitochondrial biogenesis [5,6]. Upon *Pgc1α* activation, there is an increase in the expression of uncoupling protein 1 (UCP1). UCP1 is responsible for the dissipation of the proton gradient produced by fuel oxidation, uncoupling electron transport from ATP synthesis, and leading to the production of heat inside mitochondria [7]. Besides, there is a third type of adipose tissue known as beige or brite (brown in white), which originated as a consequence of the transformation of white adipose cells into brown adipose cells [8]. This process is called browning, and it has been proposed as a possible treatment for obesity [9]. 

Lipolysis, which is defined as the hydrolysis of triacylglycerol from WAT to generate fatty acids (FAs) and glycerol, that are released into the vasculature for their use in other organs as energy substrates, was been pointed out as another mechanism for controlling obesity. When the supply of energy is necessary, those FAs undergo β-oxidation to produce energy [10]. Furthermore, lipolysis of FAs is inherent to thermogenesis activation, suggesting a close relationship between these two processes [11].

One of the mechanisms involved in the pathologies associated with obesity is the increase in reactive oxygen species (ROS) [12,13]. In general, an increase in ROS levels could act as a driver of redox signaling, yet it can ultimately trigger and promote oxidative stress if it overcomes the equilibrium between its production and degradation [14]. In addition, it was also established that mitochondrial ROS can promote thermogenesis activation [15]. Apart from mitochondria, ROS can also be generated by several enzymes, such as the family of nicotinamide adenine dinucleotide phosphate (NADPH) oxidases [16]. Within this family, NADPH oxidase 5 (NOX5) is the most recently discovered member [17,18]. The main difference with the rest of the members of the family is that NOX5 contains four EF-hands motifs, which makes NOX5 activity calcium-dependent [17]. NOX5 presents six splicing variants, but only isoforms 1 (α) and 2 (β) are catalytically active, being isoform β mainly expressed in the endothelial cells of the intima [19,20]. 

NOX enzymes are usually related to the appearance of different disorders, including metabolic ones [21], and some studies proved that NOX4 activity can modulate glucose uptake and adipocyte differentiation [22,23]. In addition, a recent study from our group suggested that mice expressing endothelial NOX5 under high-fat feeding conditions attenuate the body weight gain and lipid accumulation associated with the excess of energy intake [24]. In this context, the purpose of this study was to analyze whether endothelial NOX5 expression in mice fed with a high-fat diet (HFD) could have an effect on thermogenesis activation and lipolysis in the adipose tissue and characterize the molecular mechanisms involved. With that objective, a conditional knock-in mice model expressing NOX5 in the endothelium was used [25]. The principal findings of this work reveal that endothelial expression of NOX5 in mice fed with HFD promotes thermogenesis activation and lipolysis in adipose tissue. These results were confirmed in an in vitro model of 3T3-L1 adipocytes that were treated with conditioned media from NOX5-expressing endothelial cells incubated with glucose and palmitic acid to mimic the excess of energy supply.

## 2. Materials and Methods

### 2.1. Animals

For this study, an endothelial NOX5-β conditional knock-in mice was employed (Nox5^+/−^/Cre^+/−^). Seven-week-old male mice expressing endothelial-specific CRE recombinase (Cdh5(PAC)-CreERT2; CRE^+/−^) were employed as a control group [24,25]. NOX5-β endothelial expression was only detectable after intraperitoneal administration of tamoxifen (40 mg/kg) on 3 non-consecutive days. Mice were fed with a high-fat diet (HFD) (60% calories as fat, 20% as proteins, and 20% as carbohydrates) (OpenSource Diet Product Data-D12492, Research Diets, New Brunswick, NJ, USA) or a control diet for 10 weeks. After sacrifice, tissue samples were obtained and stored at −80 °C. All in vivo experiments were performed following the guidelines of the European Parliament Community and of the Council Directives for the care and use of laboratory animals (2016/63/EU), and all the procedures were previously approved by the University of Navarra Animal Research Review Committee, Protocol 135–16). 

### 2.2. Materials

Cell culture plasticware and cell reagents were obtained from Gibco (Thermo Fisher Scientific, Inc., Waltham, MA, USA): Dulbecco’s modified Eagle′s medium (DMEM), foetal bovine serum (FBS), 0.25% w/v trypsin-EDTA, 1% *w*/*v* penicillin-streptomycin solution, phosphate-buffered saline (PBS), Opti-MEM and Lipofectamine 3000. Reagents used for in vitro studies were purchased from Sigma Aldrich (St. Louis, MO, USA): insulin, water-soluble dexamethasone, 3-isobutyl-1-methylxanthine (IBMX), palmitic acid (PA), and glucose (Glu). Interleukin 6 (IL-6) (PMC 0064) was obtained from Gibco (Thermo Fisher Scientific, Inc., Waltham, MA, USA). General chemical reagents were purchased from Sigma Aldrich unless otherwise specified. 

### 2.3. Cell Culture

Two cell lines from American Type Culture Collection (ATCC, Manassas, VA, USA) were employed, the mouse endothelial cells bEnd.3 (CRL-2299) and mouse embryo fibroblasts 3T3-L1 (CL-173). Cells were grown at 37 °C and 5% CO_2_ in a complete medium based on DMEM supplemented with 10% FBS and 0.1% *w*/*v* penicillin-streptomycin. 3T3-L1 cells differentiation into adipocytes, and its confirmation by Oil Red staining was performed as previously described. Once 3T3-L1 cells reached confluence (day 0), cells were incubated for 48 h in DMEM supplemented with 10% FBS, insulin 1 µg/mL, dexamethasone 1 mM, and 0.5 mM IBMX. Afterward, they were incubated with DMEM containing 1 µg/mL of insulin for another 48 h. Then, cells were maintained until day 8 with DMEM supplemented with 10% FBS. On day 8, cells were considered mature adipocytes [24]. For some experiments, mature adipocytes were directly treated with recombinant IL-6 prepared at different concentrations (50, 100, 200, and 500 pg/µL).

bEnd.3 cells, previously transfected with pcDNA3.2-GFP (Control cells) or pcDNA3.2-NOX5 (NOX5 cells), were incubated in the presence of 30 mM glucose and 300 µM palmitic acid, prepared as previously described [26], to simulate obesity conditions. Briefly, a stock of 10 mM of palmitic acid was prepared in ethanol. In addition, another stock of 1.5 mM albumin free of fatty acids was prepared in a complete medium. 2 mL of each solution were mixed to obtain a 1:6 ratio (0.6 mM albumin-4 mM palmitic acid). Finally, pH was adjusted at 7.4 with NaOH. After 24 h, culture media (Glu + PA conditioned media) was collected and added to mature adipocytes for another 24 h.

### 2.4. Transient Transfection

Transfection of endothelial bEnd.3 cell line was performed with the pcDNA3.2-NOX5-β or pcDNA3.2-GFP expression plasmids. Lipofectamine 3000 Transfection Kit was employed as previously described. Transfection conditions of endothelial cells were the same as previously explained [24]. 2.5 µg of plasmid were mixed with 5 µL of Lipofectamine 3000 in 250 µL of Opti-MEM. The solution was added to the cells in presence of DMEM without FBS and antibiotics for 5 h. After the incubation period, culture medium was replaced with complete medium for 24 h (Supplementary Figure S1).

### 2.5. IL-6 ELISA

The amount of IL-6 present in the conditioned media was determined employing the ELISA kit (ab222503, Abcam, Cambridge, UK) following the manufacturer′s instructions. Briefly, cell culture media was collected and centrifuged at 2000× *g* for 10 min. Next, 50 µL of media were added to each well of a 96 well-plate and mixed with 50 µL of antibody cocktail for 1 h at room temperature. 100 µL of TMB substrate were added, and samples were incubated for 10 min in the dark. Finally, 100 µL of STOP solution were added for 1 min and the plate was read at an absorbance of 450 nm in a spectrophotometer (Thermo Fisher Scientific, Inc., Waltham, MA, USA). 

### 2.6. IL-6 Immunoprecipitation

Immunoprecipitation of IL-6 present in the conditioned media was performed with Dynabeads^TM^ protein G immunoprecipitation kit (10007D, Thermo Fisher Scientific, Inc., Waltham, MA, USA). 50 µL of dynabeads were mixed with 10 µg of IL-6 antibody (Sc-57,315; Santa Cruz Biotechnology, Dallas, TX, USA) for 10 min at room temperature. Once the antibodies were attached to the beads, 1 mL of conditioned media was added, and the mix was incubated for 30 min. After this time, tubes were placed on a magnet, and the supernatant was collected and stored at −80 °C until use. IL-6 was eluted from dynabeads using an elution buffer and stored at −20 °C for western blot analysis. 

### 2.7. Quantitative Real-Time PCR

RNA extraction was carried out using Trizol (Thermo Fisher Scientific, Inc., Waltham, MA, USA) following standard protocols. 2 µg of RNA were reverse transcribed into cDNA with M-MLV Reverse Transcriptase enzyme (Thermo Fisher Scientific, Inc., Waltham, MA, USA). Gene expression was analyzed using iQ SYBR Green Supermix kit (Bio-Rad, Hercules, CA, USA) under the following conditions: 50 °C for 2 min, 95 °C for 10 min, 40 cycles of denaturing at 95 °C during 15 s plus an annealing/extension step at 58 °C for 1 min. The reaction mix contained 2% DMSO to increase reaction performance. Glyceraldehyde 3-phosphate dehydrogenase (*Gapdh*) was used as a housekeeping gene. The specific primers (Sigma Aldrich, St. Louis, MO, USA) used are shown in Table 1.

### 2.8. Western Blot

Protein extraction was performed with RIPA buffer (25 mM Tris-HCl, 150 mM NaCl, 0.1% *w*/*v* SDS, 1% *w*/*v* sodium deoxycholate, 1% *v*/*v* IGEPAL) supplemented with a protease inhibitor cocktail (Roche, Basel, Switzerland), which contains phosphatase inhibitors such as orthovanadate and AEBSF. Western blot analysis was carried out as previously described [24]. A total of 30 µg of proteins were dissolved in loading buffer, and electrophoresis was performed at 120 V for 90 min in 10% SDS-PAGE. Transference of proteins into nitrocellulose membrane (GE Healthcare Amersham, Chicago, IL, USA) was performed at 0.35 A for 60 min. Then membranes were blocked with 5% *w*/*v* milk or 5% BSA for 1 h. After this time, membranes were incubated with primary antibodies overnight at 4 °C. Secondary antibody incubation was performed for 1 h, and results were visualized using a ChemiDOC XRS (Bio-Rad, Hercules, CA, USA) and analyzed by Quantity One 1D software (Bio-Rad, Hercules, CA, USA). Specific primary antibodies used for the study are described in Table 2. Protein levels are relative to B-Actin unless otherwise specified.

### 2.9. Statistical Analysis

Results were expressed as mean ± standard error of the mean (SEM) for those groups following a normal distribution. In the case of groups following a non-normal distribution, results were shown as a median plus confidence interval. Normality and homogeneity of variance conditions were studied by Shapiro–Wilk and Flinger test, respectively. For in vivo and in vitro data following a factorial design, comparisons among groups were performed employing two-way ANOVA (parametric) or Aligned Rank transformation ANOVA (non-parametric) test. Other conditions were evaluated with ANOVA (parametric) or Kruskal–Wallis (non-parametric) as indicated in the corresponding figure. Post-estimations were calculated using Holm correction. RStudio (RStudio Team, 2020) was used for the statistical analysis, and graphs were generated using GraphPad Prism 8 (GraphPad, San Diego, CA, USA).

## 3. Results

### 3.1. Endothelial Expression of NOX5 in Mice Fed with a High-Fat Diet Induced the Expression of Genes Related to Thermogenesis and Lipolysis

Recent work from our group showed that mice expressing NOX5 in the endothelium and exposed to a high-fat diet (HFD) showed a decrease in body weight gain (Control Cre: 14.6 g, Nox5/Cre: 10.25 g, *p* value: 0.0003) and an increase in glucose uptake (plasma glucose levels; Control Cre: 173.03 mg/mL, Nox5/Cre; 133 mg/mL, *p* value: 0.00041), as well as in the expression of genes related to glucose sensitivity in adipose tissue (Glut4 and Cav1), when compared with their control Cre fed littermates [24]. It was described in the literature that glucose uptake was necessary for BAT thermogenesis and lipid oxidation [27,28]. In this context, we decided to evaluate whether endothelial NOX5 expression could induce thermogenesis and lipolysis in WAT as an explanation for our previous results. In the present work, we continued analyzing those results employing samples from the same mice. For that purpose, Control Cre recombinase or endothelial NOX5-expressing mice were fed either a control or a HFD for 10 weeks. 

**Figure 1:**
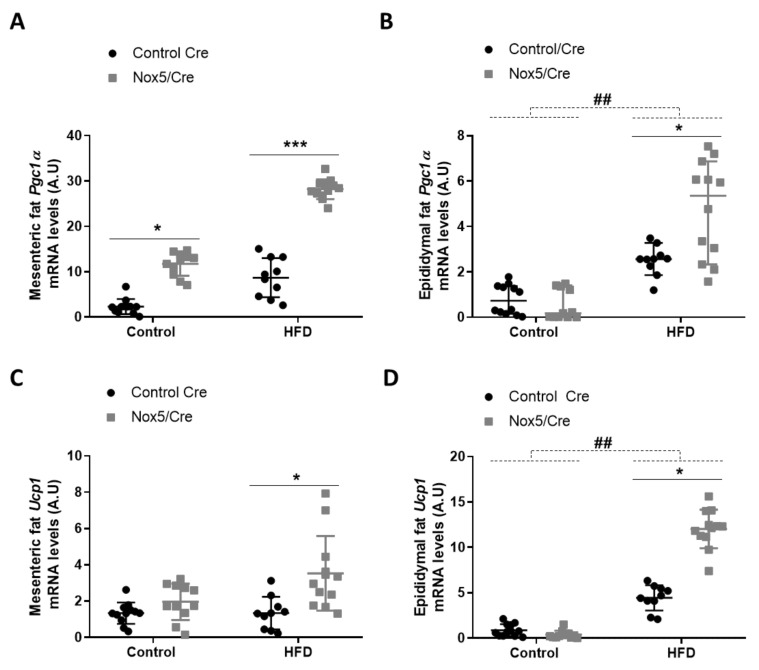
Endothelial NOX5 expression increased *Pgc1α* and *Ucp1* mRNA levels in mesenteric and epididymal fat in mice fed with a HFD for 10 weeks. *Pgc1α* mRNA levels in (**A**) mesenteric fat and (**B**) epididymal fat. *Ucp1* mRNA levels in (**C**) mesenteric fat and (**D**) epididymal fat. Control diet: control Cre (*n* = 12), Nox5/Cre (*n* = 11); HFD: control Cre (*n* = 10), Nox5/Cre (*n* = 12). Results are expressed as mean ± SEM. mRNA levels are relative to *Gapdh*. ## *p* < 0.01: diet differences (dotted lines); * *p* < 0.05, *** *p* < 0.001: genotype differences (solid lines). Statistical test used: two-way ANOVA.

To assess possible thermogenesis activation, *Pgc1α* and *Ucp1* expression was determined in mesenteric and epididymal fat tissues. There were significant differences caused by genotype in both tissues. In mesenteric fat, *p* values were <0.001 for *Pgc1α* and *Ucp1*, respectively. Mice expressing NOX5 and fed with control or HFD presented a significant increase of *Pgc1α* mRNA levels. However, only mice expressing NOX5 and fed with HFD showed significantly higher expression levels of *Ucp1* (Figure 1A,C). In epididymal fat, HFD feeding induced a significant increase of *Pgc1α* and *Ucp1*, but more interestingly, main differences in genotype (*p* value <0.001 for Pgc1α and Ucp1) provoked that among animals fed with HFD, mice expressing endothelial NOX5 experienced again a significantly higher expression of these genes than Control Cre mice (Figure 1B,D).

**Figure 2:**
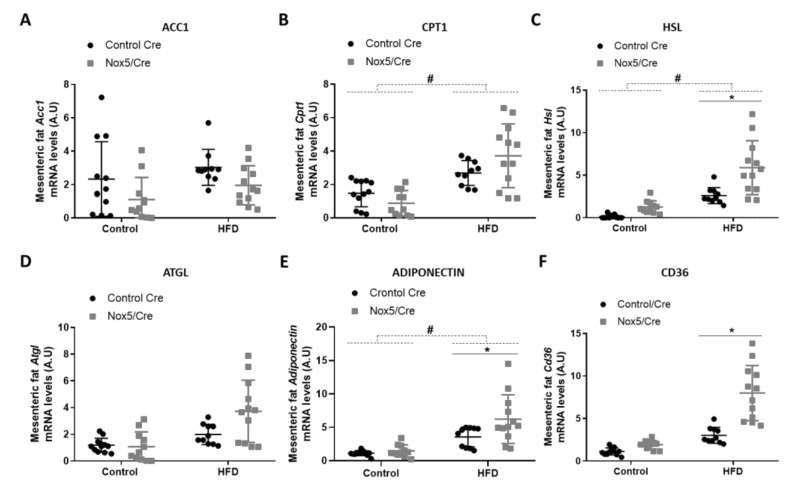
Endothelial NOX5 expression increased *Hsl*, Adiponectin and *Cd36* mRNA expression levels in mesenteric fat in mice fed a HFD for 10 weeks. (**A**) *Acc1* mRNA levels. (**B**) *Cpt1* mRNA levels. (**C**) *Hsl* mRNA levels. (**D**) *Atgl* mRNA levels. (**E**) Adiponectin mRNA levels. (**F**) *Cd36* mRNA levels. Control diet: control Cre (*n* = 12), Nox5/Cre (*n* = 11); HFD: control Cre (*n* = 10), Nox5/Cre (*n* = 12). Results are expressed as mean ± SEM. mRNA levels are relative to *Gapdh*. # *p* < 0.05: diet differences (dotted lines); * *p* < 0.05: genotype differences (solid lines). Statistical test used: two-way ANOVA.

For analyzing lipid homeostasis, we evaluated the mRNA expression levels of different genes in fat tissue samples. Specifically, six genes were studied: acetyl-CoA carboxylase 1 (*Acc1*), carnitine palmitoyltransferase 1 (*Cpt1*), hormone-sensitive lipase (*Hsl*), adipose triglyceride lipase (*Atgl*), adiponectin, and *Cd36*. An increase in the expression of these genes is associated with lipolysis upregulation except for *Acc1*. *Acc1* product (malonyl-CoA) blocks CPT1 activity, resulting in β-oxidation inhibition [29]. In mesenteric fat (Figure 2), the effect of the diet provoked that mice fed with HFD presented a significant increase in *Cpt1*, *Hsl* and adiponectin mRNA expression (Figure 2B,C,E). On the other hand, endothelial NOX5 expression in mice fed with the HFD showed a tendency to decrease *Acc1* mRNA levels (*p*-value: 0.095) and to increase mRNA levels of *Cpt1* and *Atgl* (*p* values: 0.089 and 0.076) (Figure 2A,B,D). Noteworthy, mice expressing endothelial NOX5 and fed with HFD experienced a significant increase in *Hsl*, adiponectin, and *Cd36* mRNA levels (Figure 2C,E,F).

**Figure 3:**
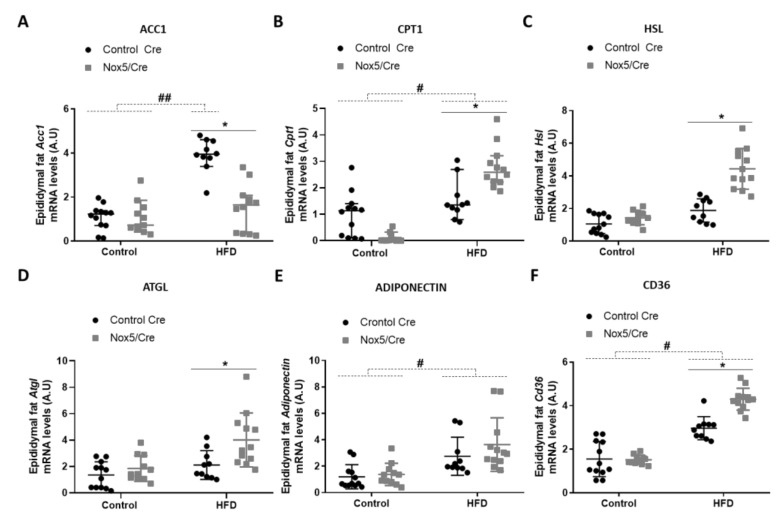
Endothelial NOX5 expression decreased *Acc1* expression and increased *Cpt1*, *Hsl*, *Atgl*, and *Cd36* mRNA expression in epididymal fat in mice fed a HFD for 10 weeks. (**A**) *Acc1* mRNA levels. (**B**) *Cpt1* mRNA levels. (**C**) *Hsl* mRNA levels. (**D**) *Atgl* mRNA levels. (**E**) Adiponectin mRNA levels. (**F**) *Cd36* mRNA levels. Control diet: control Cre (*n* = 12), Nox5/Cre (*n* = 11); HFD: control Cre (*n* = 10), Nox5/Cre (*n* = 12). Results are expressed as median with confidence interval. mRNA levels are relative to *Gapdh*. # *p* <0.05, ## *p* < 0.01: diet differences (dotted lines); * *p* < 0.05: genotype differences (solid lines). Statistical test used: Aligned Rank ANOVA.

In epididymal fat (Figure 3), the effect of the diet caused that mice fed with a HFD presented higher mRNA levels of *Acc1* and *Cd36*, and similarly to mesenteric fat in *Cpt1* and adiponectin mRNA levels (Figure 3A,B,E,F). Moreover, NOX5-expressing mice and fed with HFD showed a significant reduction of *Acc1* expression (Figure 3A) and an increase in *Cpt1*, *Hsl*, *Atgl*, and *Cd36* mRNA levels (Figure 3B,C,D,F).

Taking these results into account, it can be inferred that in mice fed with HFD, endothelial NOX5 expression could be attenuating body weight gain and lipid accumulation through activation of thermogenesis and lipolysis in the adipose tissue.

### 3.2. Endothelial NOX5 Expression Promotes an Increase of Interleukin (IL-6) Production in Adipose Tissue of Mice Fed with a High-Fat Diet and in An In Vitro Model of Endothelial bEnd.3 Cells

**Figure 4:**
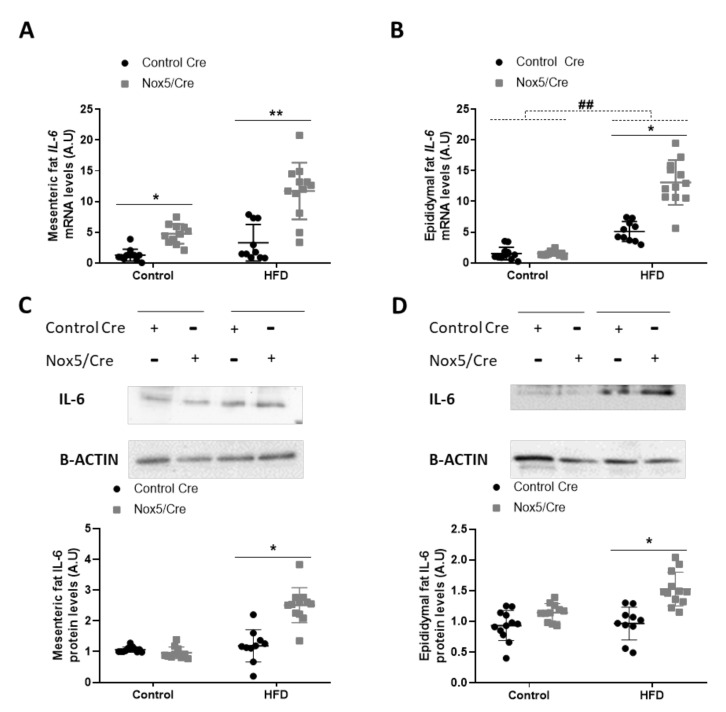
Endothelial NOX5 expression increased IL-6 expression in mesenteric and epididymal fat in mice fed with an HFD for 10 weeks. IL-6 mRNA levels in (**A**) mesenteric fat and (**B**) epididymal fat. Representative Western blots and protein levels of IL-6 in total lysates of (**C**) mesenteric fat and (**D**) epididymal fat. Control diet: control Cre (*n* = 12), Nox5/Cre (*n* = 11); HFD: control Cre (*n* = 10), Nox5/Cre (*n* = 12). Results are expressed as mean ± standard error of the mean (SEM). mRNA levels are relative to *Gapdh*. Protein levels are relative to B-ACTIN. ## *p* < 0.01: diet differences (dotted lines); * *p* < 0.05, ** *p* < 0.01: genotype differences (solid lines). The statistical test used: two-way ANOVA.

The effects observed in adipose tissue could be associated with an increase in ROS levels derived from endothelial NOX5 expression. However, as ROS production is closely related to the inflammation process, we decided to study whether the activation of NOX5 could be promoting cytokine production. The expression levels of different cytokines in the adipose tissue of mice were determined, being IL-6 the only one with a significantly augmented expression related to genotype differences: *p*-value of 0.001 for mRNA and protein levels in both mesenteric and epididymal fat. (Figure 4, Figure S2 and S3). In animals, administration of an HFD induced a significant increase in mRNA levels of IL-6 in epididymal fat (Figure 4B). Additionally, NOX5 expression had a remarkable effect in IL-6 expression in both adipose tissues, mesenteric, and epididymal, showing a significant increase, not only of mRNA, but also at protein levels when mice were fed with a HFD (Figure 4A–D). This effect of the genotype was also observed in the mesenteric fat of animals fed with a control diet, as those endothelial NOX5-expressing mice presented higher mRNA levels of IL-6 than their control Cre counterpart (Figure 4A).

**Figure 5:**
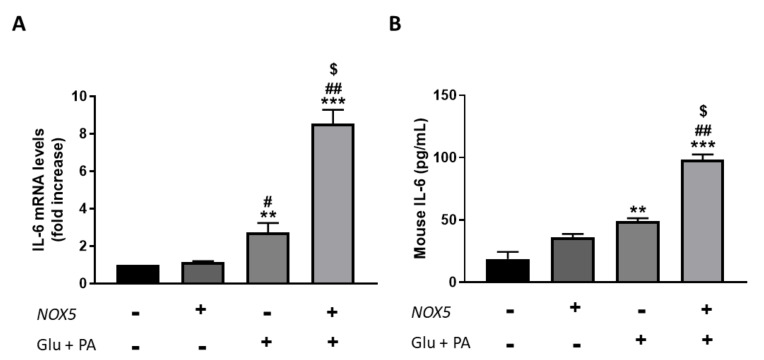
bEnd.3 cells showed higher IL-6 production when incubated for 24 h with glucose (Glu) + palmitic acid (PA) after NOX5 transfection. (**A**) IL-6 mRNA levels of endothelial bEnd.3 cells (*n* = 6). (**B**) Quantification of IL-6 concentration in culture media of endothelial bEnd.3 cells by an ELISA kit. mRNA levels are relative to *Gapdh*. Fold increase is relative to control group (Nox5(−)/Glu +PA(−)). Results are expressed as mean ± SEM; ** *p* < 0.01, *** *p* < 0.001: differences relative to Nox5(−)/Glu + PA(−); # *p* < 0.05, ## *p* < 0.01: differences relative to Nox5(+)/Glu + PA(−); $ *p* < 0.05: differences relative to Nox5(−)/Glu + PA(+). Statistical test used: ANOVA.

To confirm the results obtained in the in vivo model, we employed an in vitro cell culture model based on mouse endothelial bEnd.3 cells were transfected with a NOX5 expression plasmid for 24 h. After transfection was carried out, cells were incubated with 30 mM glucose and 300 µM palmitic acid for another 24 h to simulate the obesogenic conditions of the in vivo model. Finally, IL-6 production was determined in these cells, measuring mRNA levels as well as protein concentration in the culture media (Figure 5). In accordance with the in vivo results, those endothelial cells transfected with NOX5 and incubated with glucose and palmitic acid presented a significant increase in IL-6 mRNA levels compared to the rest of the conditions (Figure 5A). The increase in IL-6 expression was accompanied by a significantly greater concentration of IL-6 in the culture media (Figure 5B). Furthermore, this escalates in IL-6 production takes place via an NFƙB-dependent molecular pathway since an increase in the expression of the transcription factor was detected, and IL-6 production was prevented in the presence of an inhibitor of this factor (Supplementary Figure S4).

Overall, results from in vivo experiments suggest that under the influence of an HFD, endothelial NOX5 expression increases IL-6 levels in adipose tissue. Additionally, in vitro studies showed that NOX5 activity also derives in higher IL-6 production of endothelial cells in an obesogenic environment. 

### 3.3. IL-6 Production Derived from Endothelial NOX5 Expression under Obesity Conditions is Responsible of Thermogenesis Activation via STAT3 Signaling Pathway in 3T3-L1 Adipocytes

The results displayed confirm that endothelial NOX5 expression seems to promote thermogenesis activation in adipose tissue in the presence of HFD. To corroborate these results an in vitro model of bEnd.3 cells and 3T3-L1 adipocytes were employed. Endothelial cells were transfected and treated as described before. After treatment with glucose and palmitic acid, cell culture media was collected (Glu + PA conditioned media) and added to 3T3-L1 adipocytes for 24 h to simulate in vivo obesity conditions. Besides, to analyze whether IL-6 derived from NOX5 activity in mediating this signaling process, an immunoprecipitation of this cytokine from the conditioned media was carried out before its addition to the cells (IP IL-6). 

**Figure 6:**
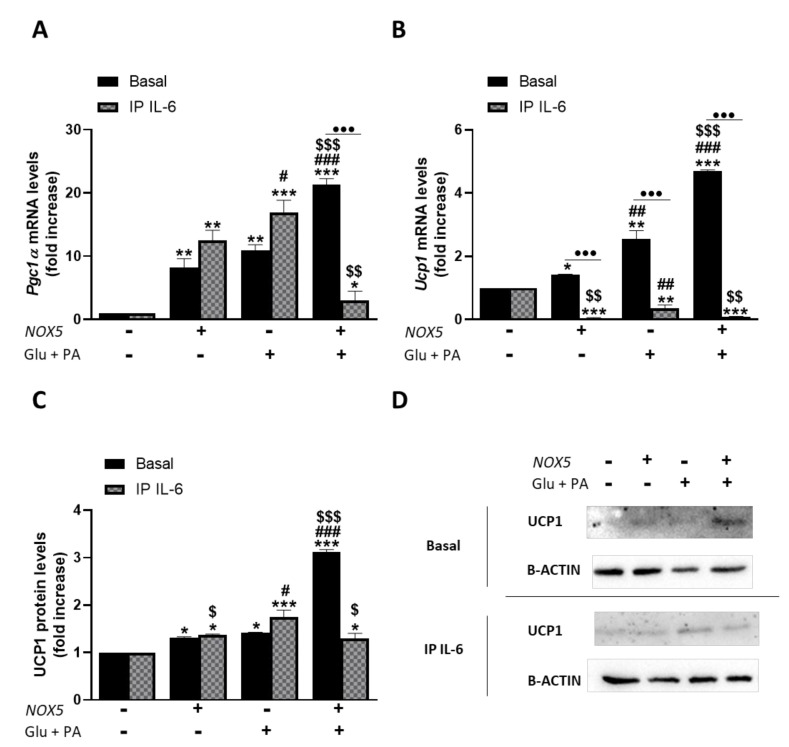
3T3-L1 adipocytes showed an increased expression of thermogenesis genes when cultured for 24 h with Glu + PA conditioned media from NOX5-expressing endothelial bEnd.3 cells. This increase is prevented when IL-6 is immunoprecipitated from the media (IP IL-6). (**A**,**B**) Pgc1α and Ucp1 mRNA levels of 3T3-L1 adipocytes (*n* = 6). (**C**) UCP1 protein levels of 3T3-L1 adipocytes (*n* = 6). (**D**) Representative images of Western blots for UCP1. mRNA levels are relative to *Gapdh*; protein levels are relative to B-ACTIN. Fold increase is relative to control group (Nox5(−)/Glu + PA(−)). Results are expressed as median plus confidence interval; * *p* < 0.05, ** *p* < 0.01, *** *p* < 0.001: differences relative to Nox5(−)/Glu + PA(−); # *p* < 0.05, ## *p* < 0.01, ### *p* < 0.001: differences relative to Nox5(+)/Glu + PA(−); $ *p* < 0.05, $$ *p* < 0.01, $$$ *p* < 0.001: differences relative to Nox5(−)/Glu + PA(+); ••• *p* < 0.001: differences relative to its basal counterpart. Statistical test used: aligned Rank ANOVA and Kruskal–Wallis.

As it can be inferred from Figure 6, Glu + PA conditioned media treatment promoted an increase of thermogenesis in mature adipocytes. Thus, in accordance with our in vivo results, endothelial NOX5 expression in the presence of glucose and palmitate induces a significantly higher expression of *Pgc1α* and *Ucp1* in adipocytes. Nevertheless, when IL-6 was immunoprecipitated from the Glu + PA conditioned media of transfected endothelial cells, the increase in mRNA levels of these genes was significantly prevented in the adipocytes. For protein levels, the results suggest that immunoprecipitation of IL-6 also prevented the increase caused by conditioned media. In addition, although NOX5 expression alone can produce a significant increase in the expression of these genes, it remained lower than the other two conditions. These results suggest the implication of endothelial IL-6 derived from NOX5 activity in the activation of adipocyte thermogenesis. Moreover, direct treatment of 3T3-L1 adipocytes with commercial IL-6 reproduced the same results obtained with the addition of Glu + PA conditioned media (Supplementary Figure S5).

**Figure 7:**
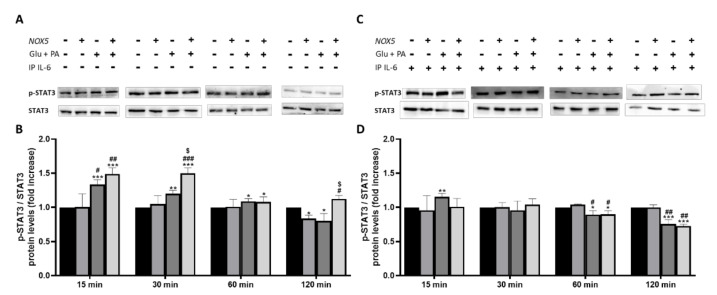
Phosphorylation levels of STAT3 are increased in 3T3-L1 adipocytes when cultured for 30 min with Glu + PA conditioned media from NOX5-expressing endothelial bEnd.3 cells (**A**,**B**). This increase is prevented when IL-6 is immunoprecipitated from the media (**C**,**D**). (**A**,**C**) Representative images of Western blots for p-STAT3 and STAT3. (**B**,**D**) p-STAT3 and STAT3 protein levels of 3T3-L1 adipocytes (*n* = 6). Results are expressed as mean ± SEM; * *p* < 0.05, ** *p* < 0.01, *** *p* < 0.001: differences relative to Nox5(−)/Glu + PA(−); # *p* < 0.05, ## *p* < 0.01, ### *p* < 0.001: differences relative to Nox5(+)/Glu + PA(−); $ *p* < 0.05: differences relative to Nox5(−)/Glu + PA(+);. Statistical test used: ANOVA.

To further confirm that IL-6 was involved in adipocyte thermogenesis activation we decided to study the levels of phosphorylated STAT3 (Figure 7). Previous studies have revealed that IL-6 can induce thermogenesis through phosphorylation of tyrosine 705 in STAT3 (p-STAT3) [30]. At basal conditions, when 3T3-L1 adipocytes were treated with Glu+ PA conditioned media, there was a significant increase of p-STAT3 at 15 and 30 min. This increase was reduced to 60 and disappeared after 120 min. Noteworthy, a further significant increase of p-STAT3 was induced when conditioned media coming from NOX5 expressing cells was added (Figure 7A,B). Interestingly, when IL-6 was immunoprecipitated from the Glu + PA conditioned media, the increase in p-STAT3 was completely prevented independently of NOX5 expression or not (Figure 7C,D).

Taken together, these observations indicate that Glu + PA conditioned media from NOX5-expressing endothelial cells might increase thermogenesis in 3T3-L1 adipocytes through an IL-6/p-STAT3 dependent molecular pathway. 

### 3.4. IL-6 Production Derived from Endothelial NOX5 Expression under Obesity Conditions is Responsible of Lipolysis Activation via AMPK Signaling Pathway in 3T3-L1 Adipocytes

**Figure 8:**
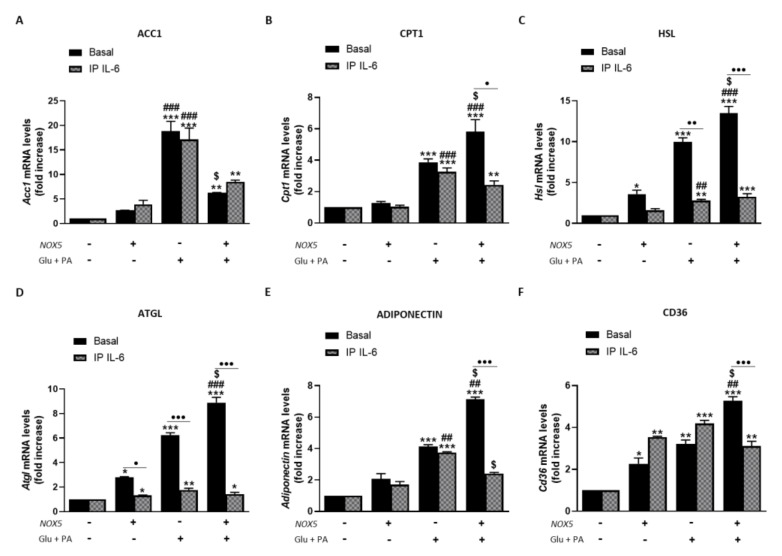
3T3-L1 adipocytes showed increased lipolysis when cultured for 24 h with Glu + PA conditioned media from NOX5-expressing endothelial bEnd.3 cells. This increase is prevented when IL-6 is immunoprecipitated from Glu + PA conditioned media. (**A**) *Acc1* mRNA levels in 3T3-L1 adipocytes. (**B**) *Cpt1* mRNA levels in 3T3-L1 adipocytes. (**C**) *Hsl* mRNA levels in 3T3-L1 adipocytes. (**D**) *Atgl* mRNA levels in 3T3-L1 adipocytes. (**E**) Adiponectin mRNA levels in 3T3-L1 adipocytes. (**F**) *Cd36* mRNA levels in 3T3-L1 adipocytes. mRNA levels are relative to *Gapdh*. Fold increase is relative to control group (Nox5(−)/Glu + PA(−)). Results are expressed as median plus confidence interval; * *p* < 0.05, ** *p* < 0.01, *** *p* < 0.001: differences relative to Nox5(−)/Glu + PA(−); ## *p* < 0.01, ### *p* < 0.001: differences relative to Nox5(+)/Glu + PA(−); $ *p* < 0.05: differences relative to Nox5(−)/Glu + PA(+); • *p* < 0.05, •• *p* < 0.01, ••• *p* < 0.001: differences relative to its basal counterpart. Statistical test used: aligned Rank ANOVA.

Resembling the thermogenesis process, endothelial NOX5 expression seems to induce lipolysis in adipose tissue of mice fed with HFD. In this sense, we analyzed the role of IL-6 in this process using the in vitro model (Figure 8). When 3T3-L1 adipocytes were cultured with Glu + PA conditioned media for 24 h, there was a significant increase in mRNA levels of all lipolytic genes analyzed that was augmented for *Cpt1*, *Hsl*, *Atgl*, adiponectin, and *Cd36* when NOX5 was also expressed (Figure 8A–F). On the contrary, in the case of *Acc1*, the expression of NOX5 in the endothelial cells provoked a significantly lower increase in its mRNA levels (Figure 8A). Moreover, NOX5 alone promoted an increase in the expression levels of *Hsl*, *Atgl*, and *Cd36*. Still, this increase was lower than the induced by the rest of the conditions. In addition, IL-6 immunoprecipitation from Glu + PA conditioned media of NOX5 expressing cells significantly prevented the increase in mRNA expression levels of all genes except for *Acc1* (Figure 8A–F). Finally, direct treatment of 3T3-L1 cells with commercial IL-6 originated similar changes in expression levels as those obtained with the addition of Glu + PA conditioned media of NOX5 expressing cells conditioned media (Supplementary Figure S6).

These results suggest that endothelial NOX5 expression could induce lipolysis in adipocytes under obesity conditions via IL-6.

**Figure 9:**
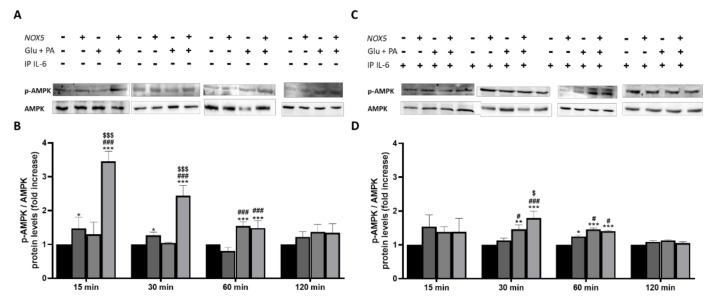
Phosphorylation levels of AMPK are increased in 3T3-L1 adipocytes when cultured for 15, 30 and 60 min with Glu + PA conditioned media from NOX5-expressing endothelial bEnd.3 cells (**A**,**B**). This increase is prevented when IL-6 is immunoprecipitated from the media (**C**,**D**). (**A**,**C**) Representative images of Western blots for p-AMPK and AMPK. (**B**,**D**) p-AMPK and AMPK protein levels of 3T3-L1 adipocytes (*n* = 6). Results are expressed as mean ± SEM; * *p* < 0.05, ** *p* < 0.01, *** *p* < 0.001: differences relative to Nox5(−)/Glu + PA(−); # *p* < 0.05, ### *p* < 0.001: differences relative to Nox5(+)/Glu + PA(−); $ *p* < 0.05, $$$ *p* < 0.001: differences relative to Nox5(−)/Glu + PA(+); Statistical test used: ANOVA.

Other studies demonstrated that the activation of fatty acid oxidation through phosphorylation of the cellular energy sensor adenosine monophosphate-activated protein kinase (AMPK) is mediated by IL-6 [31]. Considering the crucial role played by IL-6 in the present work, we decided to analyze the phosphorylation levels of AMPK (p-AMPK). p-AMPK levels were determined in 3T3-L1 adipocytes when treated with Glu + PA conditioned media before and after IL-6 immunoprecipitation (Figure 9). As it can be observed, at 15, 30, and 60 min, there was a significant increase of p-AMPK levels when adipocytes were treated with Glu + PA conditioned media from NOX5-expressing endothelial cells (Figure 9A,B). However, when IL-6 was removed from media, levels of p-AMPK did not reach the same extent (Figure 9C,D).

Considering this information, it seems that NOX5-expressing endothelial, in the presence of high concentrations of glucose and fats, regulates lipolysis in adipocytes. This regulation appears to be mediated through an IL-6/p-AMPK dependent manner.

## 4. Discussion

Obesity has become a global health issue over the last few years. Bodyweight gain is tightly regulated by synchronized effects on energy intake and expenditure. Subsequently, several studies have tried to stimulate energy expenditure through heat production, pursuing a therapeutic perspective [32]. Within this context, the browning of WAT into beige adipose tissue has acquired great interest. Non-shivering thermogenesis of BAT and beige adipose tissue promotes an increase in lipolysis of triglycerides and β-oxidation of FAs, resulting in a decrease of adiposity [33,34,35]. Redox signaling, derived from mitochondrial ROS, is involved in the activation of this process [36,37]. Remarkably, although NADPH oxidases (NOXs) are one of the family of enzymes directly involved in ROS homeostasis, their role in the regulation of thermogenesis has not been fully elucidated [38]. Generally, these professional oxidases have been involved in the development of different pathologies such as hypertension or diabetes [39]. However, the role of the most recently discovered member of this family, NOX5, is not completely understood, mainly due to its evolutionary loss from the genome of rodents. A recent study from our group has proved that in mice, endothelial NOX5 expression under obesity conditions attenuates body weight gain, lipid accumulation and increases glucose uptake [24]. To gain more insight into this effect, we explored the possible involvement of endothelial NOX5 expression in the browning process of white adipose tissue and in the lipolytic activity of mice fed with an HFD and the molecular mechanisms associated.

It has been previously described that moderate levels of mitochondrial ROS increase *Ucp1* expression, promoting thermogenesis, which results in energy expenditure as heat [36]. However, an excessive amount of ROS leads to oxidative stress impairing adipocyte’s function and suppressing thermogenesis [40]. In the present work, endothelial NOX5 expression stimulates non-shivering thermogenesis by upregulating *Pgc1α* and *Ucp1* in both mesenteric and epididymal fat in mice fed with an HFD (Figure 1). In addition, it has also been proved that ROS can induce fat mobilization of adipose tissue by lipolysis activation [41]. As a result, these mice that present an increase in thermogenesis also show an upregulation of some genes associated with lipolysis (Figure 2 and Figure 3). Specifically, mice expressing endothelial NOX5 and fed with an HFD experience an increase in the expression of *Cpt1*, *Hsl*, *Atgl*, Adiponectin, and *Cd36*. These findings are in accordance with studies that proved how the expression of these genes was necessary for thermogenesis activation [42,43,44,45,46]. It is particularly interesting the increase in the expression of adiponectin since it has been pointed out that, in the liver, adiponectin induces a better insulin response. Furthermore, in the muscle, adiponectin induces phosphorylation of ACC1, which results in CPT1 activation and induction of β oxidation [47,48]. On the other hand, knock out mice for adiponectin was shown to present alterations in the regulation of gluconeogenesis [49]. We can conclude that, in our model, endothelial expression of NOX5 in mice fed with an HFD, might be modulating body weight gain and lipid accumulation through non-shivering thermogenesis and enhanced lipolysis. Thus, endothelial Nox5 activity would be producing redox signaling as an adaptive response to the insult promoted by the HFD. Considering these results, it would also be interesting for future works to analyze the role of leptin. Since its discovery, leptin has been considered as a possible alternative for the treatment of obesity due to its ability to stimulate lipolysis and raising body temperature [50,51]. Additionally, there are studies correlating the presence of ROS with leptin [52]. 

Recent studies have shown that BAT thermogenesis is regulated through the secretion of different cytokines [53]. Moreover, Issa et al., demonstrated that cytokines induce lipolysis in 3T3-L1 adipocytes by increasing NOX3 expression and superoxide production [54]. Therefore, we evaluated the expression of different cytokines in the adipose tissue of mice fed with a control diet or an HFD. Only IL-6 levels were significantly increased by endothelial expression of NOX5 in both mesenteric and epididymal WAT of mice fed with an HFD (Figure 4) (S.M.2–3). On the other hand, an in vitro model of NOX5-transfected murine endothelial cells, cultured in the presence of high concentrations of glucose and palmitic acid, showed a similar increase of IL-6 expression (Figure 5). At a molecular level, the in vitro model also proved that, in endothelial cells, the increase of IL-6 is mediated through the activation of the transcription factor Nf-ƙB (S.M.4). Similarly, the group of Dr. Zheng has recently described that oxidative stress induced by NOX5 causes inflammation via Nf-ƙB/IL-6 [55]. Additionally, IL-6 has been found to be specifically induced as a response to a disturbed redox status [56], and in cerulein-stimulated pancreatic acinar cells, NADPH oxidase activity induced Nf-ƙB activation and IL-6 expression in AR42j cells [57].

Previous studies have shown that IL-6 can modulate body weight gain, lipolysis and induce thermogenesis in obese mice [58,59]. Thus, we further explored in our in vitro model, based on 3T3-L1 adipocytes treated with conditioned media coming from NOX5-expressing endothelial cells exposed to high concentration of glucose and palmitic acid (Glu + PA conditioned media), whether the increased levels of IL-6 derived from NOX5 expression could explain the effects in the thermogenic process and lipid metabolism markers observed in our animal model. For that purpose, IL-6 was immunoprecipitated from the Glu + PA conditioned media, and the effect of this media on the adipocytes was analyzed. Those adipocytes treated with complete conditioned media upregulated the genes related to thermogenesis (*Pgc1α* and *Ucp1*). However, IL-6 withdrawal by immunoprecipitation significantly prevented the overexpression of these genes (Figure 6). Moreover, looking into the molecular mechanisms involved in this process, we have also confirmed that the modulation of the thermogenic process takes place through phosphorylation of STAT3, as previously described [30,60]. When 3T3-L1 were treated with Glu + PA conditioned media, there was an increase of phosphorylated STAT3, that was prevented upon IL-6 immunoprecipitation (Figure 7). Additionally, treatment of adipocytes with different concentrations of recombinant IL-6 replicated the results obtained with the Glu + PA conditioned media (S.M.5). Therefore, it seems that endothelial NOX5 expression, under conditions of high concentration of glucose and palmitic acid, induces the secretion of IL-6, which in turn triggers thermogenesis in adipocytes by upregulating *Pgc1α* and *Ucp1* expression, through a STAT3 phosphorylation-induced mechanism. 

In addition, the effect of IL-6 on the mentioned lipid markers was also analyzed. Adipocytes cultured with Glu + PA conditioned media coming from NOX5-expressing endothelial cells experienced an increase in the expression of genes associated with lipolysis. In full accordance with the previous results, the immunoprecipitation of IL-6 from the media prevented the upregulation of these genes (Figure 8). As it happened with thermogenesis, direct treatment of adipocytes with recombinant IL-6 also increases the expression of the lipolytic genes (S.M.6). It has been previously described in the literature that IL-6 can induce lipolysis through the phosphorylation of AMP-activated protein kinase (AMPK), a central regulator of energy homeostasis that generally suppresses anabolic ATP-consuming pathways while stimulating catabolic ATP-generating pathways [61]. In this context, AMPK modulates lipid metabolism reducing lipid storage through phosphorylation of several substrates in distinct pathways that collectively promote fatty acid oxidation while suppressing fatty acid and cholesterol biosynthesis [62]. In our in vitro model, we confirmed that IL-6 released from Glu + PA conditioned media due to NOX5 activity promotes the phosphorylation of AMPK in the adipocytes, activation that is prevented by the withdrawal of the cytokine from the media (Figure 9). Thus, we report here that endothelial NOX5 expression under obesity conditions stimulates lipolysis by promoting AMPK phosphorylation mediated by IL-6 production. 

Finally, the higher metabolic activity of BAT correlates with higher glucose metabolism [63]. It has been previously described in the literature that in skeletal muscle, acute IL-6 exposure induces the phosphorylation of STAT3 and subsequent AMPK activation, which in turn increases GLUT4 translocation to the plasma membrane [64]. On the other hand, Wang et al., demonstrated a relationship between the administration of IL-6 and caveolin 1 (*Cav1*) expression in vascular endothelial cells [65]. Our previous work proved that endothelial NOX5 expression in the presence of an HFD enhanced glucose uptake characterized by an increase in Glut4 and Cav1 expression [24]. As a result, we wanted to confirm whether the increase in insulin sensitivity that we had detected was also dependent on the endothelial secretion of IL-6. Once again, in our in vitro model, the immunoprecipitation of the cytokine from Glu + PA conditioned media coming from NOX5-expressing endothelial cells reduced glucose uptake, modified lipid accumulation, and prevented the increase of *Glut4* and *Cav1* expression that was observed in those 3T3-L1 adipocytes when the complete non-immunoprecipitated conditioned media was added (Supplementary Figure S7).

**Figure 10:**
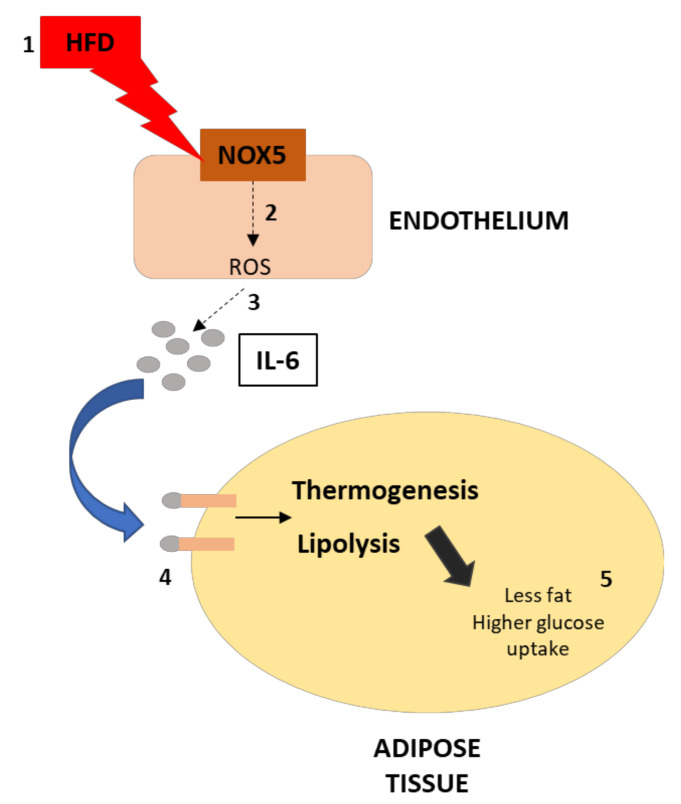
Graphical scheme of the experimental design and principal findings. (**1**) The effect of HFD over endothelial cells increase NOX5 activity, which increases ROS production (**2**). This increase in ROS production leads to a higher production of IL-6 in the endothelium (**3**). As a consequence, the activity of IL-6 over adipose tissue results in thermogenesis and lipolysis activation (**4**). Finally, the upregulation of these processes derives in less fat accumulation and higher glucose uptake of the adipose tissue (**5**).

In summary, we have found that the expression of endothelial NOX5 in mice under obesity conditions increases the production of IL-6 in the endothelium. This cytokine causes an upregulation of thermogenesis and lipolysis in neighboring white adipose tissue via STAT3 and AMPK, respectively. Consequently, these results suggest that the endothelial expression of NOX5 may contribute to regulating weight gain, lipid homeostasis, and glucose uptake through these two processes (Figure 10). The translation of the present results to humans should be made with caution, taking into consideration the differences between humans and mice in energy metabolism and thermogenesis, as well as the relatively reduced amount of BAT in humans.

## Figures and Tables

**Table 1:** Sequences of primers used for quantitative real-time PCR.

Gene	Accession Number		Primers
Interleukin 6 (*Il-6*)	NM.031168.2	Forward	5′-CAGAATTGCCATTGCACAAC-3′
		Reverse	5′-AGTTGCCTTCTTGGGACTGA-3′
		Forward	5′-TACGCAGGTCGAACGAAACT-3′
Peroxisome proliferative activated receptor, gamma, coactivator 1 alpha (*Pgc1α*)	NM.008904.2	Reverse	5′-ATCCAACCTGCACAAGTTCC-3′
Uncoupling protein 1 (*Ucp1*)	NM.009463.3	Forward	5′-GGCCTCTACGACCTCAGTCCA-3′
		Reverse	5′-TAAGGCCGGCTGAGATCTTGT-3′
Acetyl-Coenzyme A carboxylase α (*Acc1*)	NM.133360.2	Forward	5′-GCCTCTTCCTGACAAACGAG-3′
		Reverse	5′-TGACTGCCGAAACATCTCTG-3′
Carnitine palmitoyltransferase 1 (*Cpt1*)	NM.013495.2	Forward	5′-CAGAGGATGGACACTGTAAA-3′
		Reverse	5′-CGGCACTTCTTGATCAAGCC-3′
Hormone sensitive lipase (*Hsl*)	NM.010719.5	Forward	5′-GCTGGGCTGTCAAGCACTGT-3′
		Reverse	5′-ATGGCAGCCTACCCAGTTAC-3′
Adipose triglyceride lipase (*Atgl*)	NM.001163689.1	Forward	5′-CAACGCCACTCACATCTACGG-3′
		Reverse	5′-TCACCAGGTTGAAGGAGGGAT-3′
Adiponectin	NM.009605.5	Forward	5′-TGGATCTGACGACACCAAAA-3′
		Reverse	5′-ATCCAACCTGCACAAGTTCC-3′
*Cd36*	NM.01159558.1	Forward	5′-TTGCTGCCTTCTGAAATGTG-3′
		Reverse	5′-GCAGAATCAAGGGAGAGCA-3′
Glyceraldehyde-3-phosphate dehydrogenase (*Gapdh*)	NM.008969.4	Forward	5′-ATGACAACTTTGTCAAGCTCATTT-3′
		Reverse	5′-GGTCCACCACCCTGTTGCT-3′

**Table 2:** Antibodies used for Western Blot analysis.

Primary Antibody	Specie	Dilution	Commercial House	Secondary Antibody	Dilution
Interleukin 6 (IL-6)	Mouse	1:1000	Sc-57315, Santa Cruz Biotechnology	Anti-mouse (NA931V, GE Healthcare)	1:4000
Uncoupling protein 1 (UCP1)	Rabbit	1:500	ab10983, Abcam	Anti-rabbit (NA934V, GE Healthcare)	1:1000
p-STAT3 (Ser 727)	Rabbit	1:500	#9134s, Cell Signaling		1:1000
STAT3	Rabbit	1:1000	#9132, Cell Signaling		1:2000
p-AMPKα (Thr 172)	Rabbit	1:500	#2531s, Cell Signaling		1:1000
AMPKα	Rabbit	1:1000	#2532, Cell Signaling		1:2000
B-ACTIN	Mouse	1:10,000	A1978, Sigma-Aldrich	Anti-mouse (NA931V, GE Healthcare)	1:10,000

## Data Availability

The data presented in this study are available on request from the corresponding author.

## Supplementary Materials

The following are available online at https://www.mdpi.com/article/10.3390/antiox11010030/s1, Figure S1: NOX5 transfection of bEnd.3 cell line; Figure S2: mRNA levels of different cytokines in the mesenteric fat of mice; Figure S3: mRNA levels of different cytokines in the epididymal fat of mice; Figure S4: Endothelial bEnd.3 cells presented higher expression of NFƙB when cells were transfected with Nox5 expression plasmid and were incubated with glucose and palmitic acid. Inhibition of NFƙB (PDTC) prevented IL-6 production compared to conditioned media; Figure S5: 3T3-L1 adipocytes presented higher expression of Pgc1α and Ucp1 when they were incubated for 24 h with different concentrations of recombinant IL-6; Figure S6: 3T3-L1 adipocytes presented higher expression of lipolytic genes when they were incubated for 24 h with different concentrations of recombinant IL-6; Figure S7: 3T3-L1 adipocytes presented reduced lipid accumulation and glucose uptake as well as lower expression of Glut4 and Cav1 when IL-6 was immunoprecipitated from Glu + PA conditioned media of NOX5 expressing bEnd.3 endothelial cells.
