# Supplementary material for: Endothelial NOX5 Expression Modulates Thermogenesis and Lipolysis in Mice Fed with a High-Fat Diet and 3T3-L1 Adipocytes through an Interleukin-6 Dependent Mechanism
Source: Antioxidants (Basel). 2021 Dec 24;11(1):30. doi: 10.3390/antiox11010030 (PMC8772862; doi:10.3390/antiox11010030)
Supplement: Supplementary file 1 [file antioxidants-11-00030-s001.zip › antioxidants-1482082-supplementary.pdf]

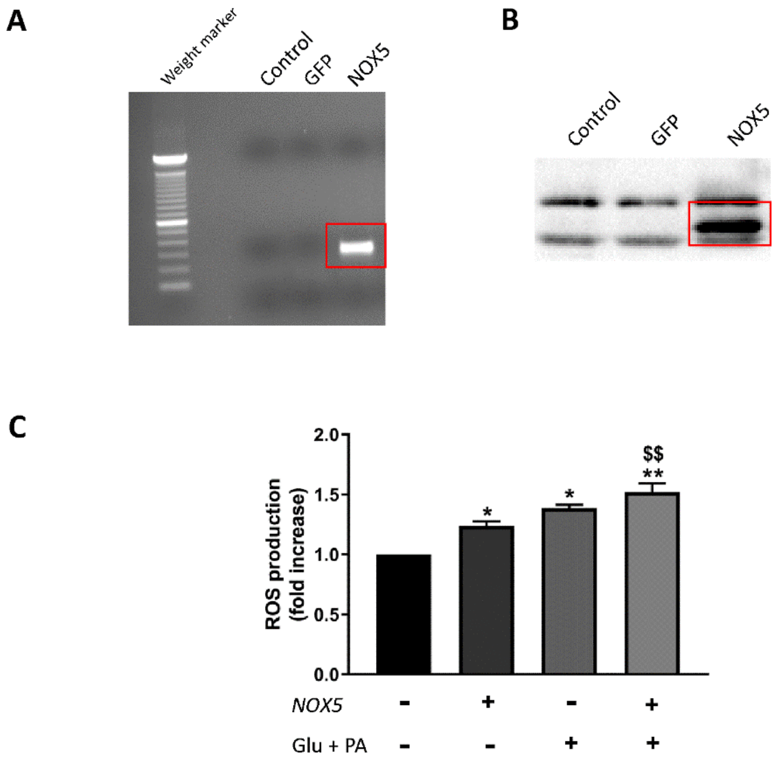

Figure S1. ***NOX5* transfection of bEnd.3 cell line.** (**A**) Conventional PCR for Nox5 detection after 24 hours of transfection. (**B**) Representative Western Blot of NOX5 after 24 hours of transfection. (**C**) Measurement of NOX5 activity by Amplex Red after 24 hours of transfection. Cells were treated for 5 hours with 30 mM glucose and 300 μM palmitic acid to promote NOX5 activity. Amplex Red assay was performed following manufacturer instructions (#A12222, Thermo Fisher Scientific). Fold increase is relative to the control group (Nox(-)/Glu+PA(-)). Values are expressed as mean ± SEM. *p<0.05: differences relative to Nox(-)/Glu+PA(-); $$p<0.01: differences relative to Nox(-)/Glu+PA(+). Statistical test used: ANOVA.

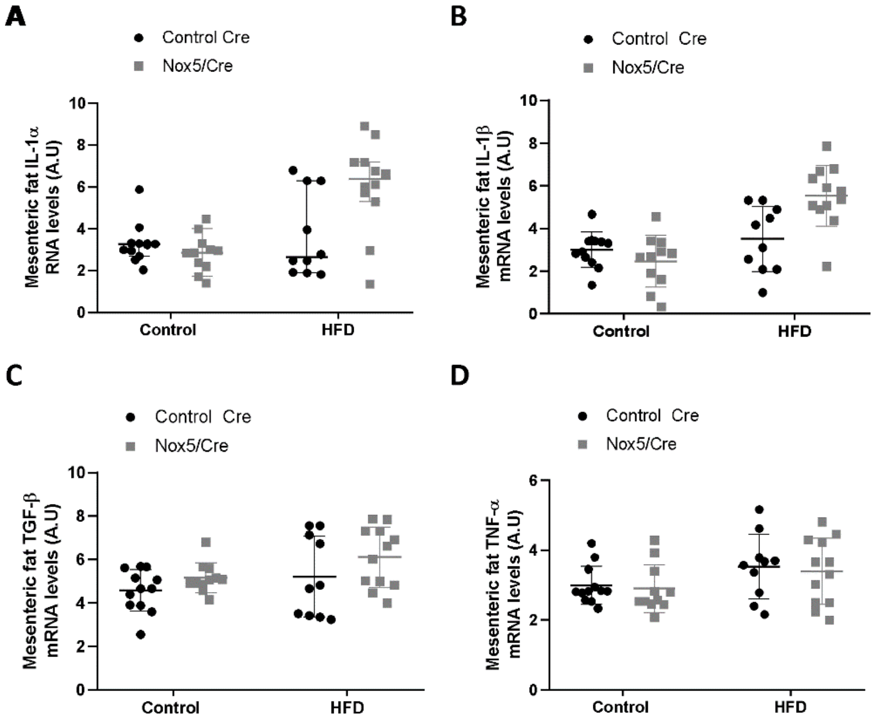

Figure S2. mRNA levels of different cytokines in the mesenteric fat of mice. (**A**) mRNA levels of *Il-1α*. (**B**) mRNA levels of *Il-1β*. (**C**) mRNA levels of *Tgf-β*. (**D**) mRNA levels of *Tnf-α*. Control diet: Control Cre (n=12),

Nox5/Cre (n=11); High-fat diet: Control Cre (n=10), Nox5/Cre (n=12). Results are expressed as mean ± SEM. mRNA levels are relative to *Gapdh*.

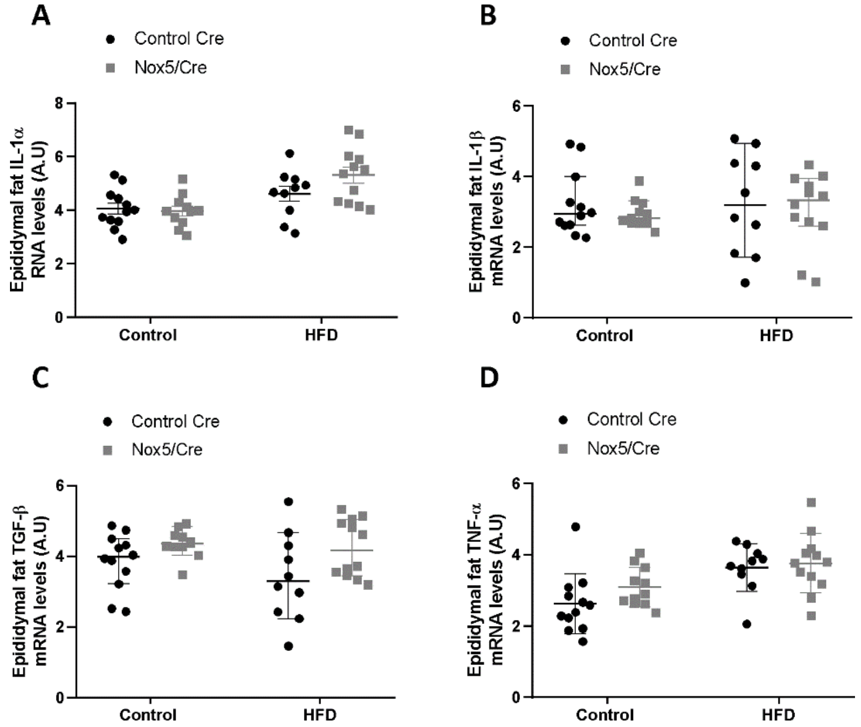

Figure S3. mRNA levels of different cytokines in the epididymal fat of mice. (**A**) mRNA levels of *Il-1α*. (**B**) mRNA levels of *Il-1β*. (**C**) mRNA levels of *Tgf-β*. (**D**) mRNA levels of *Tnf-α*. Control diet: Control Cre (n=12), Nox5/Cre (n=11); High-fat diet: Control Cre (n=10), Nox5/Cre (n=12). Results are expressed as median with confidence interval. mRNA levels are relative to *Gapdh*.

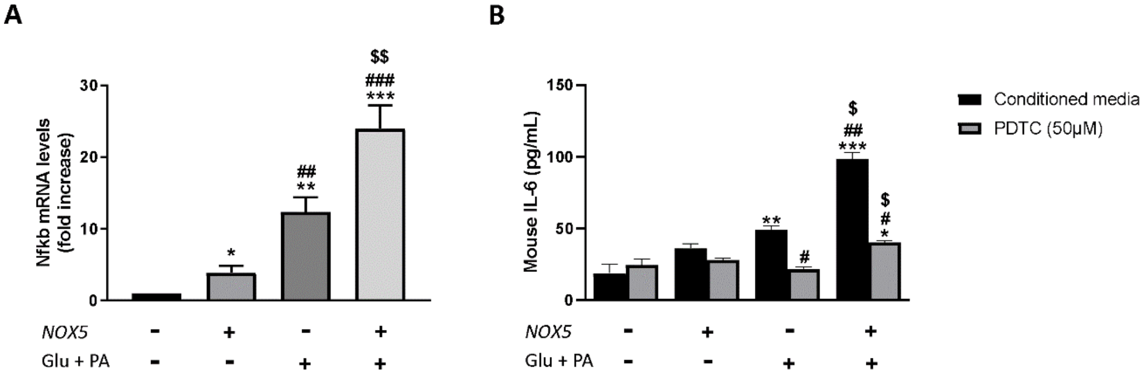

Figure S4. Endothelial bEnd.3 cells presented higher expression of NFκB when cells were transfected with Nox5 expression plasmid and were incubated with glucose and palmitic acid. Inhibition of NFκB (PDTC) prevented IL-6 production compared to conditioned media. (**A**) mRNA levels of *NFκB*. (**B**) Concentration of IL-6 in the culture media of bEnd.3 cells in presence or absence of an inhibitor (PDTC). N=6. mRNA levels are relative to *Gapdh*. Fold increase is relative to group (Nox(-)/Glu + PA(-)). Results are expressed as mean ± SEM. * $p<0.05$, ** $p<0.01$, *** $p<0.001$: differences relative to Nox(-)/Glu + PA (-); # $p<0.05$, ## $p<0.01$, ### $p<0.001$: differences relative to Nox(+)/Glu + PA(-); $ $p<0.05$, $$ $p<0.01$: differences relative to Nox(-)/Glu + PA(+). Statistical test used: ANOVA.

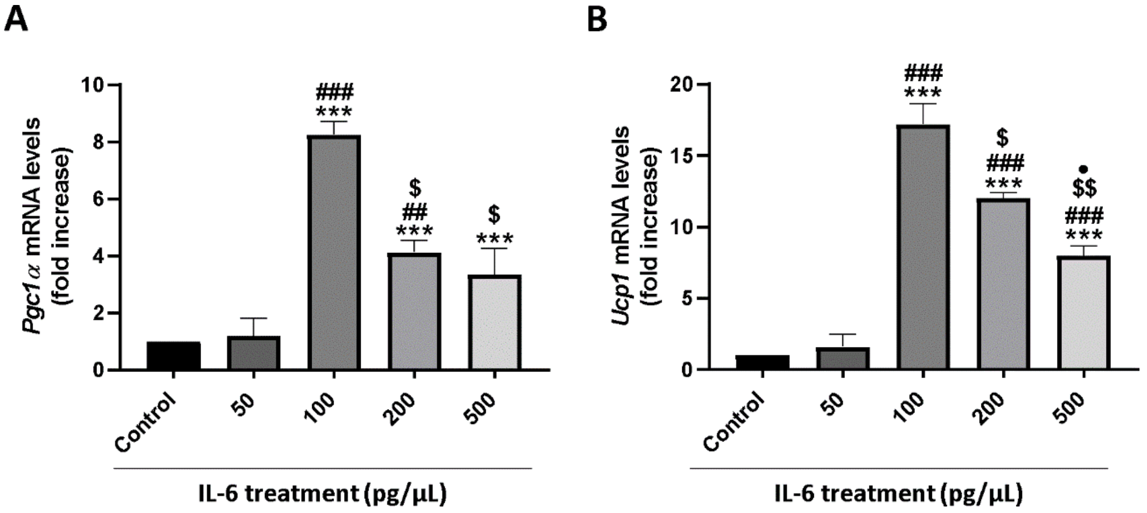

Figure S5. 3T3-L1 adipocytes presented higher expression of *Pgc1α* and *Ucp1* when they were incubated for 24 hours with different concentrations of recombinant IL-6. (**A**) mRNA levels of *Pgc1α*. (**B**) mRNA levels of *Ucp1*. N=6. mRNA levels are relative to *Gapdh*. Fold increase is relative to control group. Results are expressed as mean ± SEM. *** p<0.001: differences relative to Control; ## p<0.01, ###p<0.001: differemces relative to 50 pmol; $ p<0.05, $$ p<0.01: differences relative to 100 pmol; • p<0.05: differences relative to 200 pmol. Statistical test used: ANOVA.

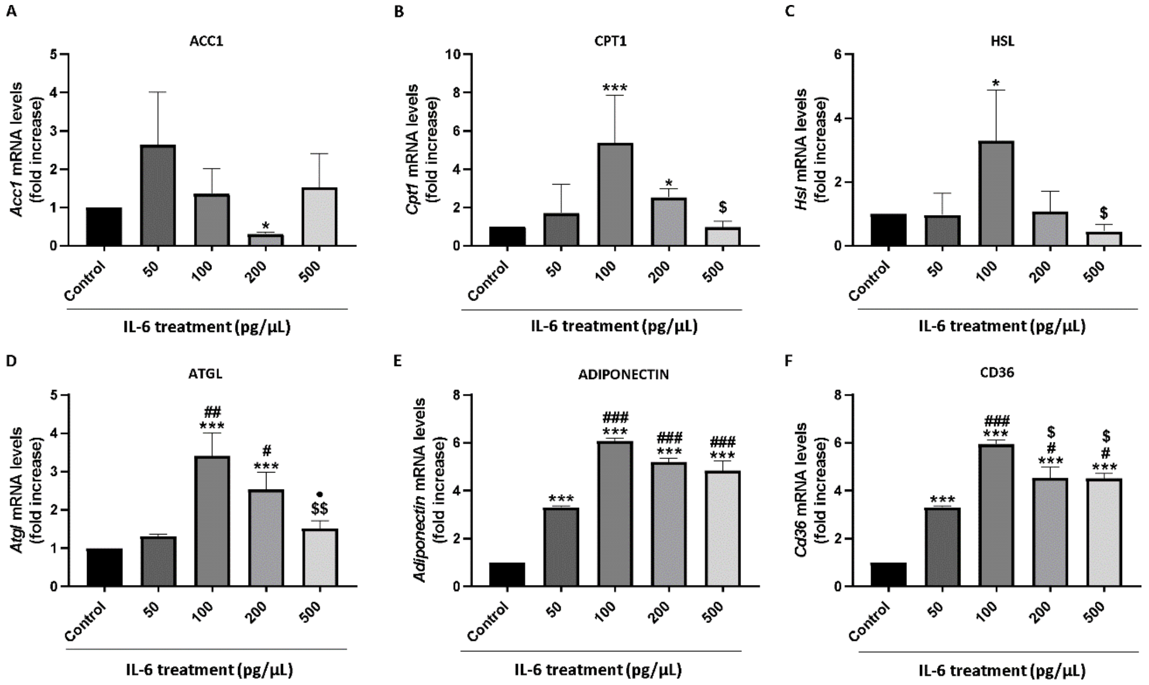

Figure S6. 3T3-L1 adipocytes presented higher expression of lipolytic genes when they were incubated for 24 hours with different concentrations of recombinant IL-6. (**A**) mRNA levels of *Acc1*. (**B**) mRNA levels of *Cpt1*. (**C**) mRNA levels of *Hsl*. (**D**) mRNA levels of *Atgl*. (**E**) mRNA levels of *Adiponectin*. (**F**) mRNA levels of *Cd36*. N=6. mRNA levels are relative to *Gapdh*. Fold increase is relative to control group. Results are expressed as mean ± SEM. * p<0.05, *** p<0,001: differences relative to Control; # p<0.05, ## p<0.01, ###p<0.001: differences relative to 50 pmol; $ p<0.05, $$ p<0.01: differences relative to 100 pmol; • p<0.05: differences relative to 200 pmol. Statistical test used: ANOVA.

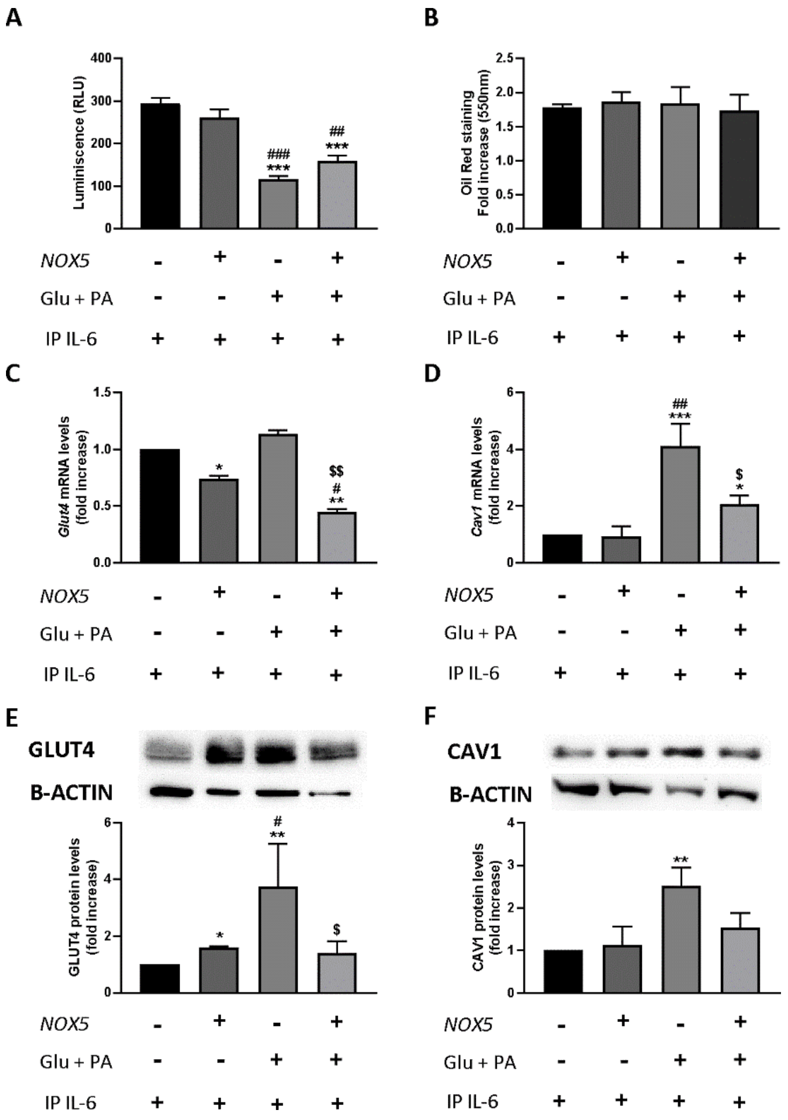

Figure S7. Lipid accumulation, glucose uptake and expression of Glut4 and Cav1 was prevented in 3T3-L1 adipocytes when incubated for 24 h with Glu + PA conditioned media of NOX5 expressing endothelial bEnd.3 cells previously immunoprecipitated for IL-6. (**A**) Glucose uptake quantification of 3T3-L1 adipocytes through luminescence after stimulation with 100 nM insulin. (**B**) Oil Red quantification at 550 nM. (**C**) mRNA levels of *Glut4.* (**D**) mRNA levels of *Cav1.* (**E**) Representative images and protein levels of GLUT4. (**F**) Representative images and protein levels of CAV1. N=6. mRNA levels are relative to *Gapdh*. Protein levels are relative to B-ACTIN. Fold increase is relative to group (Nox(-)/Glu + PA(-)). Results are expressed as mean ± SEM. * $p<0.05$ ** $p<0.01$, *** $p<0.001$: differences relative to Nox(-)/Glu + PA (-); # $p<0.05$, ## $p<0.01$, ### $p<0.001$: differences relative to Nox(+)/Glu + PA(-); $ $p<0.05$, $$ $p<0.01$: differences relative to Nox(-)/Glu + PA(+). Statistical test used: ANOVA.
